# Targeting the Calcineurin Homologous Protein 1 (CHP1)-Transmembrane Protein 87A (TMEM87A) mechanosensing complex: a druggable vulnerability in metastatic ovarian cancer

**DOI:** 10.1186/s43556-026-00487-4

**Published:** 2026-06-08

**Authors:** Ming-Zhu Jin, Heng-An Liu, Wen Di

**Affiliations:** 1 Department of Obstetrics and Gynecology, Renji Hospital, Shanghai Jiao Tong University School of Medicine, Shanghai, People’s Republic of China; 2 Shanghai Key Laboratory of Gynecologic Oncology, Renji Hospital, Shanghai Jiao Tong University School of Medicine, Shanghai, People’s Republic of China; 3 Mater Research Institute, The University of Queensland, Brisbane, QLD Australia

**Keywords:** Calcineurin homologous protein 1 (CHP1), TMEM87A, Mechanosensitive, Sodium gluconate, Drug repurposing, Ovarian cancer

## Abstract

Despite advances in treatment, metastatic high-grade serous ovarian carcinoma (HGSC) remains largely incurable, and the molecular machinery enabling tumor cells to survive detachment and disseminate intraperitoneally remains poorly understood. HGSC metastasis requires tumor cells to evade anoikis during detachment from the fallopian tube epithelium. Through integrative analysis of hyaluronic acid (HA) metabolism-associated genes in transcriptomic datasets, calcineurin homologous protein 1 (CHP1) was identified as a novel mediator of HGSC metastasis. Mechanistically, CHP1 ablation disrupted spheroid morphogenesis in 3D models, inducing aberrant multi-spheroid formation and reduced structural integrity, with impaired cell extrusion from mosaic spheroids. Bioinformatic and experimental validation revealed that CHP1 interacted with the mechanosensitive channel transmembrane protein 87A (TMEM87A), forming a putative mechanosensing complex that may regulate metastasis via the YAP–GPC6–WNT5A/Hedgehog signaling axis; knockout of either gene downregulated *WNT5A/GPC6* and inhibited WNT5A/Hedgehog pathways. In vivo, *CHP1* or *TMEM87A* knockout significantly suppressed orthotopic tumor growth and metastasis in NSG mice, indicating a role in overall tumor progression beyond metastasis alone. Drug repurposing identified sodium gluconate (a TMEM87A modulator) as a therapeutic candidate: low-dose treatment (1 mg/kg, weekly) significantly reduced tumor burden by disrupting CHP1-TMEM87A binding and inhibiting downstream Hedgehog/PTCH1 signaling, as the cellular thermal shift assay (CETSA), microscale thermophoresis (MST), surface plasmon resonance (SPR), and co-immunoprecipitation. Clinically, high *CHP1/TMEM87A* expression exhibited stage-dependent prognostic significance (favorable in early-stage, unfavorable in late-stage HGSC), as assessed by Kaplan–Meier survival analysis of publicly available datasets. These findings suggest that the CHP1-TMEM87A complex may serve as a mechanosensitive regulator of HGSC metastasis and propose gluconate-based targeting as a promising therapeutic strategy.

**Supplementary Information:**

The online version contains supplementary material available at 10.1186/s43556-026-00487-4.

## Introduction

Ovarian cancer remains a leading cause of gynecological cancer mortality [1], largely due to its highly aggressive nature and unique pattern of intraperitoneal dissemination. Although the advent of novel therapeutic options, such as poly ADP-ribose polymerase (PARP inhibitors), has significantly improved the five-year survival rate for ovarian cancer, the majority of patients eventually develop recurrent and treatment-resistant disease. Accumulating research has increasingly highlighted the critical role of the physical properties of the tumor microenvironment (TME), especially the increased stiffness of the extracellular matrix (ECM), in driving cancer cell invasion, metastasis, and drug resistance. These physical and biomechanical features are collectively referred to as the mechanical microenvironment, which we previously described as a specialized component of the TME [2]. Cancer cells adapt to this harsh mechanical microenvironment and promote malignant progression by sensing these mechanical force signals and converting them into biochemical signals. However, the specific molecular mechanisms, particularly the key factors, remain underexplored and urgently need to be elucidated.

A hallmark of HGSC progression is the ability of tumor cells to detach from the primary site, survive as multicellular aggregates (spheroids) in the peritoneal fluid, and implant on distant peritoneal surfaces, a process critically dependent on resistance to anoikis. During this transcoelomic dissemination, tumor cells must sense and adapt to dramatic changes in their mechanical microenvironment, including loss of matrix anchorage and exposure to fluid shear stress [3–5]. Despite the clinical importance of this metastatic route, the molecular mechanisms by which HGSC cells maintain spheroid integrity and mechanosensory competence during peritoneal dissemination remain incompletely understood.

Hyaluronic acid (HA), a major component of the ECM, plays a crucial role in the tumor microenvironment, including regulating cell adhesion and migration. Elevated hyaluronidase activity has been observed in ovarian cancer, suggesting that cancer cells exploit inherent tissue properties to facilitate invasion and metastasis [6]. In a screen based on HA metabolism, we identified calcineurin homologous protein 1 (*CHP1*) as a potential key regulator. CHP1 is a widely expressed multifunctional protein that plays important roles in various physiological and pathological processes, particularly in cellular pH homeostasis, ion transport, and signal transduction. Previous studies have shown that CHP1 acts as an auxiliary subunit for the Na^+^/H^+^ exchanger (NHE), directly binding to and significantly enhancing the transport activity of NHE1, which is essential for maintaining intracellular pH homeostasis and sodium ion concentration [7–9]. Furthermore, CHP1 regulates glycerolipid synthesis in the endoplasmic reticulum by binding to and activating glycerol-3-phosphate acyltransferase 4 (GPAT4). The N-myristoylation of CHP1 is critical for its interaction with GPAT4 and its function [10]. In this context, we hypothesize that CHP1 may be a key regulator in the cellular response to external mechanical stimuli. This study demonstrates that CHP1, through a specific interaction with the mechanosensitive protein transmembrane protein 87 A (TMEM87A) [11, 12], co-mediates the response of ovarian cancer cells to mechanical microenvironmental signals. This discovery provides a novel perspective on the mechanisms of ovarian cancer metastasis, and offers a potential avenue for developing targeted therapeutic strategies.

Mechanosensing, the ability of cells to detect and respond to physical forces, is increasingly recognized as a critical driver of cancer progression [13, 14]. Mechanosensitive ion channels, including PIEZO1/2 and TMEM87A, transduce extracellular mechanical cues into intracellular biochemical signals that regulate cell survival, migration, and invasion [15, 16]. In the context of HGSC, tumor cells must navigate a mechanically dynamic microenvironment during peritoneal dissemination [4, 17], yet the molecular machinery governing this process remains incompletely understood. Here, we show that the CHP1-TMEM87A complex may constitute a previously unrecognized putative mechanosensing hub that coordinates spheroid integrity and metastatic competence, and that this complex can be pharmacologically targeted by sodium gluconate.

In the present study, we demonstrate that CHP1 and TMEM87A form a functional complex that governs spheroid integrity, mechanosensing, and metastatic competence in HGSC. Genetic ablation of either component disrupts spheroid morphogenesis, impairs GPC6-WNT5A/Hedgehog signaling, and suppresses orthotopic tumor growth and metastasis in vivo. Furthermore, drug repurposing identifies sodium gluconate as a low-dose therapeutic agent capable of disrupting the CHP1-TMEM87A interaction and suppressing tumor progression, providing a novel and clinically translatable strategy for targeting this mechanosensing vulnerability in metastatic ovarian cancer.

## Results

### CHP1 is upregulated in HGSC and promotes metastasis

**Fig. 1:**
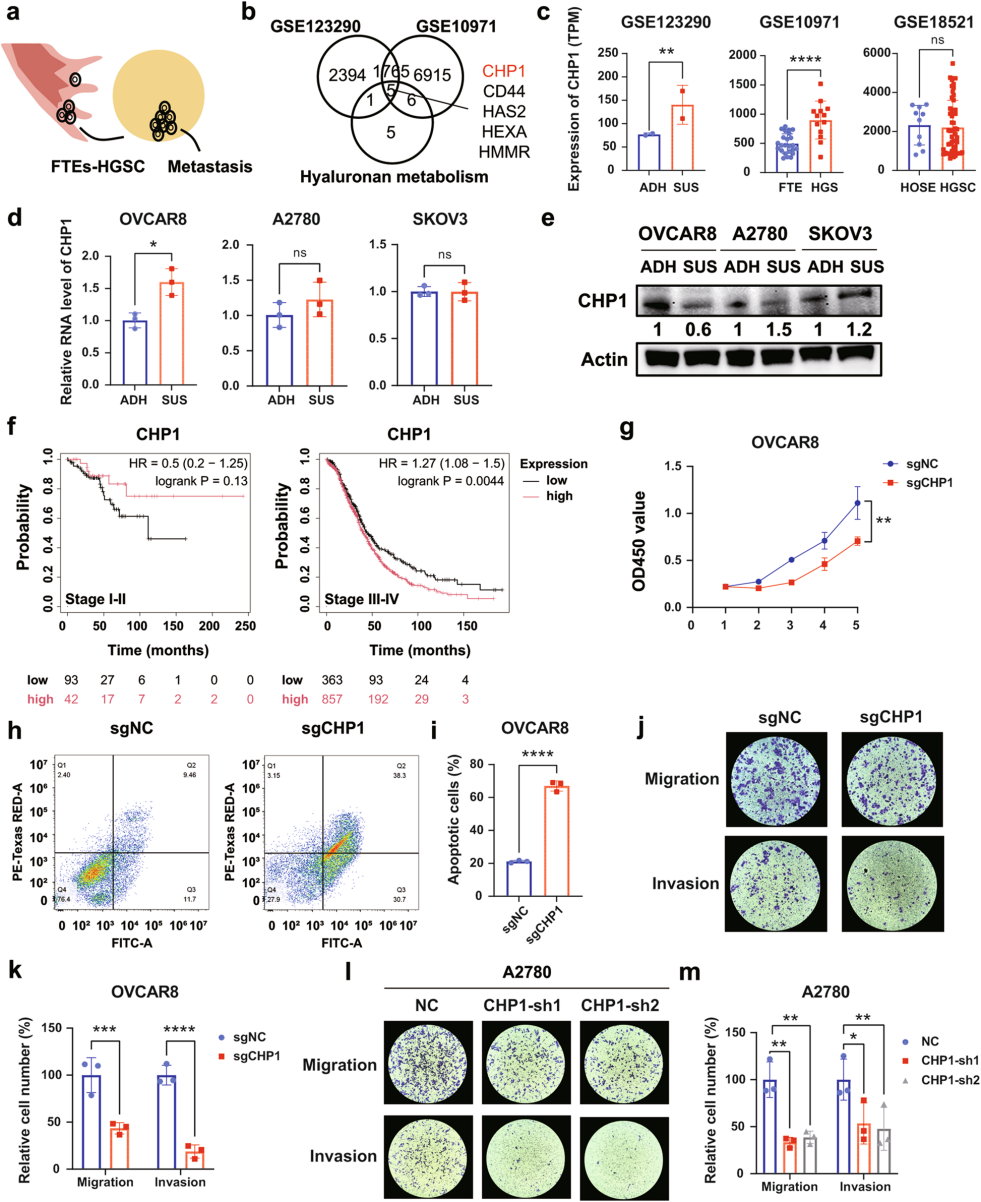
CHP1 is upregulated in HGSC and promotes metastasis. **a** Schematic diagram of HGSC origin from fallopian tube epithelium (FTEs) and intraperitoneal metastasis. **b** Venn diagram showing the intersection of upregulated genes from GSE123290, GSE10971, and HA metabolism-associated genes, identifying *CHP1* as a candidate. **c ***CHP1* expression (TPM) in adherent vs. suspended cells (GSE123290), normal fallopian tube epithelium vs. high-grade serous carcinoma (GSE10971), and normal human ovarian surface epithelium (HOSE) vs. HGSC (GSE18521). **d** RT-qPCR analysis of *CHP1* mRNA expression in adherent (ADH) vs. suspended (SUS) OVCAR8, A2780, and SKOV3 cells (n = 3 independent experiments). **e** Western blot analysis of CHP1 protein expression in adherent vs. suspended OVCAR8, A2780, and SKOV3 cells; Actin serves as loading control. **f** Kaplan–Meier survival analysis of CHP1 expression in stage I–II and stage III–IV ovarian cancer patients. **g** CCK-8 proliferation assay of OVCAR8-*sgNC* and *sgCHP1* cells (n = 3 independent experiments). **h** Representative flow cytometry scatter plots (Annexin V/PI staining) of apoptosis in OVCAR8-*sgNC* and *sgCHP1* cells under suspension conditions. **i** Quantification of apoptosis rate (% Annexin V-positive cells) in OVCAR8-*sgNC* and *sgCHP1* cells under suspension conditions (n = 3 independent experiments). **j** Representative images of migration and invasion assays for OVCAR8-*sgNC* and *sgCHP1* cells. **k** Quantification of relative cell number (%) in migration and invasion assays for OVCAR8-*sgNC* and *sgCHP1* cells (n = 3 independent experiments). **l** Representative images of migration and invasion assays for A2780 cells with NC, CHP1-sh1, and CHP1-sh2. **m** Quantification of relative cell number (%) in migration and invasion assays for A2780 cells (n = 3 independent experiments). * *P* < 0.05; ** *P* < 0.01; *** *P* < 0.001; **** *P* < 0.0001

A significant proportion of HGSC arise from the distal fallopian tube epithelium. Tumor cells progressing from serous tubal intraepithelial carcinomas (STICs) to invasive disease must detach from the basement membrane and evade anoikis [18] (Fig. 1a). Given the importance of HA in cell adhesion, migration, and signaling, we investigated the potential role of HA metabolism-associated genes in ovarian cancer metastasis. The HA metabolism-associated gene list was defined by intersecting differentially expressed genes from GSE123290 (adherent vs. suspended cells) [19] and GSE10971 (normal fallopian tube epithelium vs. ovarian carcinoma) [20, 21] with HA-interacting molecules curated from GeneCards (search term: ‘hyaluronic acid metabolism’). This intersection yielded five candidate genes: *CD44*, *HAS2*, *HEXA*, *HMMR*, and *CHP1* (Fig. 1b; Table S1). All five HA metabolism-associated genes were significantly upregulated in suspended cells compared to adherent cells. Moreover, their expression levels were also elevated in ovarian carcinomas relative to normal fallopian tube epithelium or borderline tissues, suggesting that their upregulation may contribute to disease progression. Four of the five genes (*CD44*, *HAS2*, *HEXA*, *HMMR*) are already well-established key regulators of HA metabolism with extensive prior research in cancer metastasis. The inclusion of *CHP1* among HA metabolism related genes may be attributed to its role as a chaperone of NHE1. Midgley et al. previously demonstrated that the inhibition of NHE1, CD44v7/8, or HYAL2 hindered BMP7-driven HA internalization and the subsequent interaction with CD44 [22]. The activation of NHE1 and HYAL2 promoted HA degradation upon low shear stress [23]. However, the expression of *CHP1* had a weak correlation with other critical HA metabolism-related genes (Fig. S1a). *CHP1* was significantly upregulated under forced suspension and in HGSC compared to normal fallopian tube epithelium, but not when compared to human ovarian surface epithelial (Fig. 1c). *CHP1* RNA and protein expression were examined in three ovarian cancer cell lines (OVCAR8, A2780, SKOV3) after 48 h of forced suspension culture (Fig. 1d-e). In OVCAR8 cells, *CHP1* RNA expression was significantly upregulated, whereas CHP1 protein expression was downregulated. Meanwhile, in A2780 cells, *CHP1* RNA expression showed only a modest, non-significant upregulation. The discordance between *CHP1* mRNA upregulation and protein downregulation in OVCAR8 cells under suspension is an intriguing observation. This may reflect post-transcriptional regulation, such as increased protein degradation or altered translational efficiency under anchorage-independent conditions [24]. Similar mRNA-protein discordances have been reported for other mechanosensitive proteins under mechanical stress and may represent a compensatory feedback mechanism [25]. The differential response across cell lines likely reflects the high heterogeneity inherent in ovarian cancer subtypes. Although its expression was not associated with the overall survival in ovarian cancer patients across all stages, higher expression levels of *CHP1* were associated with improved prognosis in early-stage patients (stage I–II; n = 135, HR = 0.50, logrank P = 0.13), but poorer outcomes in late-stage patients (stage III–IV; n = 1,220, HR = 1.27, logrank P = 0.0044), based on Kaplan–Meier analysis using the KM-plotter database (Fig. 1f). These findings suggest that CHP1 may play a context-dependent role in cancer metastasis.

OVCAR8 was primarily used for subsequent experiments in this study. We generated *CHP1*-knockout OVCAR8 cells with CRISPR/Cas9. Sanger sequencing confirmed successful ablation with a homozygous 17-bp frameshift deletion (non-triplet multiple), and the WB validated a complete depletion of *CHP1* (Fig. S1b). *CHP1* knockout also significantly reduced cell proliferation, as assessed by the CCK-8 assay, and while increased apoptosis under suspension conditions, as shown by Annexin V/PI staining (Fig. 1g–i). The knockdown of *CHP1* inhibited cell migration and invasion in both OVCAR8 and A2780 cells (Fig. 1j–m).

### CHP1 loss disrupts spheroid morphogenesis and impairs 3D tumor organization

**Fig. 2:**
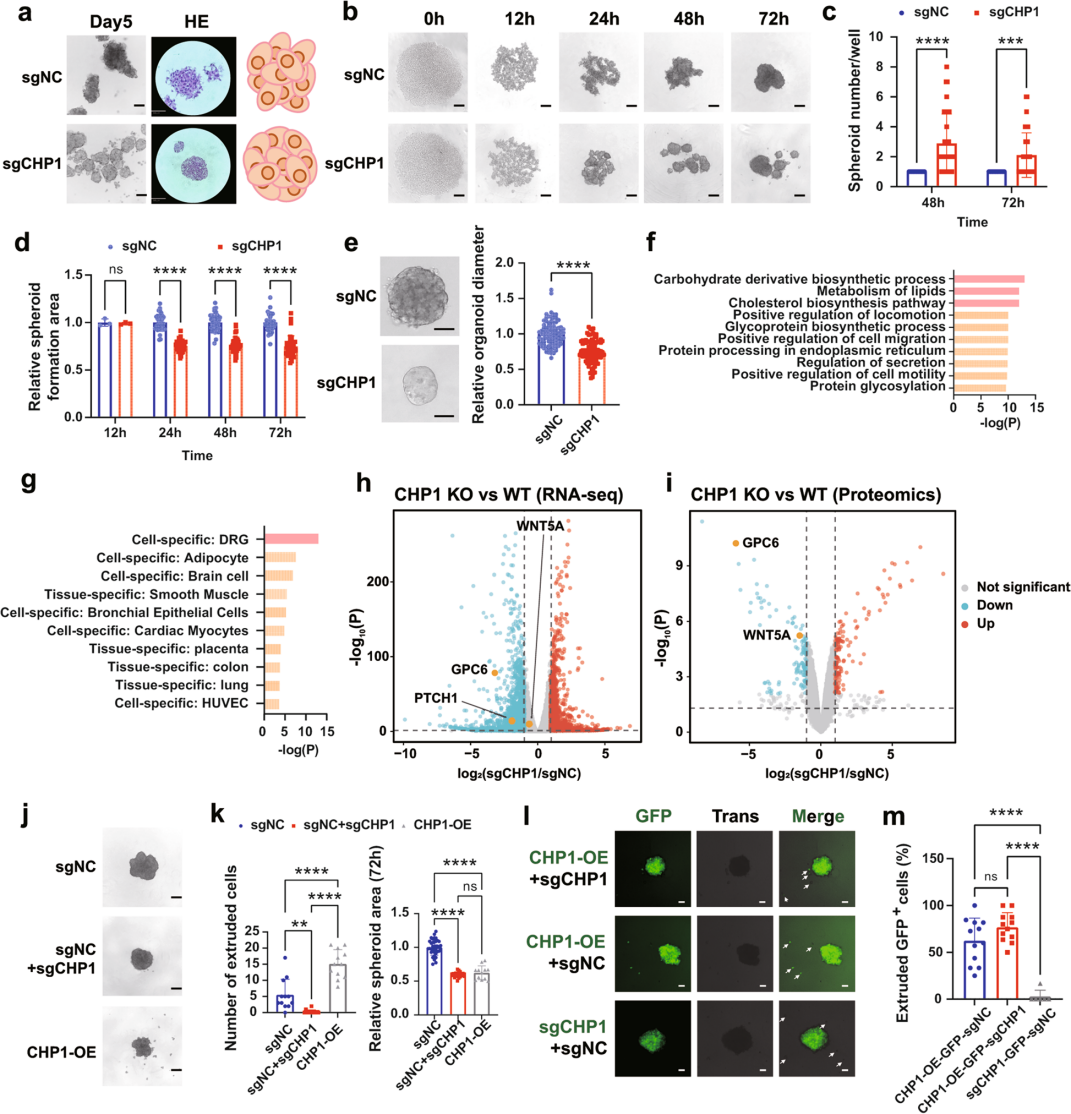
CHP1 loss disrupts spheroid morphogenesis and impairs 3D tumor organization. **a** Representative images of spheroid formation of OVCAR8-*sgNC* and *sgCHP1* in low-attachment 24-well plates, corresponding H&E staining, and schematic illustration. Scale bar, 50 μm. **b** Time-course images of OVCAR8-*sgNC* and *sgCHP1* spheroid formation (3,000 cells/well) in ULA 96-well plates with centrifugation at 0, 12, 24, 48, and 72 h. Scale bar, 50 μm. **c** Spheroid number per well at 48 and 72 h (n = 36 wells per group). **d** Relative spheroid formation area at 12, 24, 48, and 72 h (n = 36 wells per group). **e** Representative images of organoids derived from OVCAR8-*sgNC* and *sgCHP1* cells and quantification of relative organoid diameter (sgNC n = 90, sgCHP1 n = 102). Scale bar, 50 μm. **f** GO enrichment analysis of downregulated genes in *sgCHP1* vs. *sgNC* cells. **g** Tissue-specific gene enrichment analysis of downregulated genes in *sgCHP1* vs. *sgNC* cells. **h** Volcano plot showing downregulation of *GPC6* and *PTCH1* mRNA expression upon *CHP1* knockout (RNA-seq). **i** Volcano plot showing downregulation of GPC6 and WNT5A protein expression upon CHP1 knockout (proteomics). **j** Representative images of mosaic spheroids formed by OVCAR8-*sgNC*, *sgNC* + *sgCHP1*, and *CHP1-OE* cells. Scale bar, 50 μm. **k** Quantification of number of extruded cells (left) and relative spheroid area at 72 h (right) for *sgNC* (n = 36), *sgNC* + *sgCHP1* (n = 24), and *CHP1-OE* (n = 12) mosaic spheroids. **l** Representative fluorescence images of mosaic spheroids composed of *CHP1-OE*-GFP + *sgCHP1*, *CHP1-OE*-GFP + *sgNC*, and *sgCHP1*-GFP + *sgNC* cells. Arrows indicate extruded GFP^+^ cells. Scale bar, 50 μm. **m** Quantification of extruded GFP + cells (%) in mosaic spheroids: *CHP1-OE*-GFP + sgNC (n = 12), *CHP1-OE*-GFP + *sgCHP1* (n = 12), and *sgCHP1*-GFP + *sgNC* (n = 6). * *P* < 0.05; ** *P* < 0.01; *** *P* < 0.001; **** *P* < 0.0001

To better depict tumor structure, a three-dimensional (3D) spheroid model was employed. Upon suspension, OVCAR8-*sgNC* cells tended to form spheroids with irregular margin, while OVCAR8-*sgCHP1* primarily generated small spheroids with regular margin, which was similar to that observed in melanoma when over-expression of NHE1 brought about a more irregularly shaped spheroid [26] (Fig. 2a). Using U-bottom 96-well ULA plates with centrifugation, *sgCHP1* cells spontaneously generated multiple small spheroids per well across all seeding densities (1,000/3,000/8,000 cells/well) without additives (Fig. 2b-d). The phenomenon, referred to as "multiple spheroids in a single droplet", was previously reported in HEK293 spheroid culture with macromolecule supplement such as dextran (DEX), polyethylene glycol (PEG), and methylcellulose, indicating that viscosity modification and secondary macromolecule effects can induce particle and cellular aggregation [27, 28]. The size of organoids cultured in Matrigel was also significantly reduced following *CHP1* knockdown (Fig. 2e).

RNA-sequencing showed that *CHP1* knockdown inhibited TGF-β and Hippo signaling pathways, and revealed enrichment in carbohydrate derivative biosynthesis, lipid metabolism, and cell migration/motility pathways (Fig. 2f). Tissue-specific gene set enrichment analysis identified strong associations with dorsal root ganglion (DRG)-related pathways, suggesting sensory-like adaptations and increasing interest in mechanosensitive pathways (Fig. 2g). *CHP1*-knockout cells showed significant downregulation of Wnt signaling effector with a decrease in *GPC6* and *WNT5A*, aligning with literature where GPC6 loss inhibits WNT5A-driven migration (Fig. 2h–i) [29]. *CHP1* overexpression decreased spheroid diameter but increased the number of extruded cells (Fig. 2j–k).

To better understand the relationship between CHP1 expression and cell–cell organization, mosaic spheroids were created among CHP1-OE (GFP-labeled), *sgNC*, and *sgCHP1* cells. Dissociated cells were consistently observed around the mosaic spheroids and invariably possessed higher CHP1 expression (Fig. 2l–m). These collective findings suggest that CHP1 plays a role in regulating mechanical responses and spheroid formation.

### TMEM87A is a key interacting partner of CHP1 and shares its mechanosensitive phenotype

**Fig. 3:**
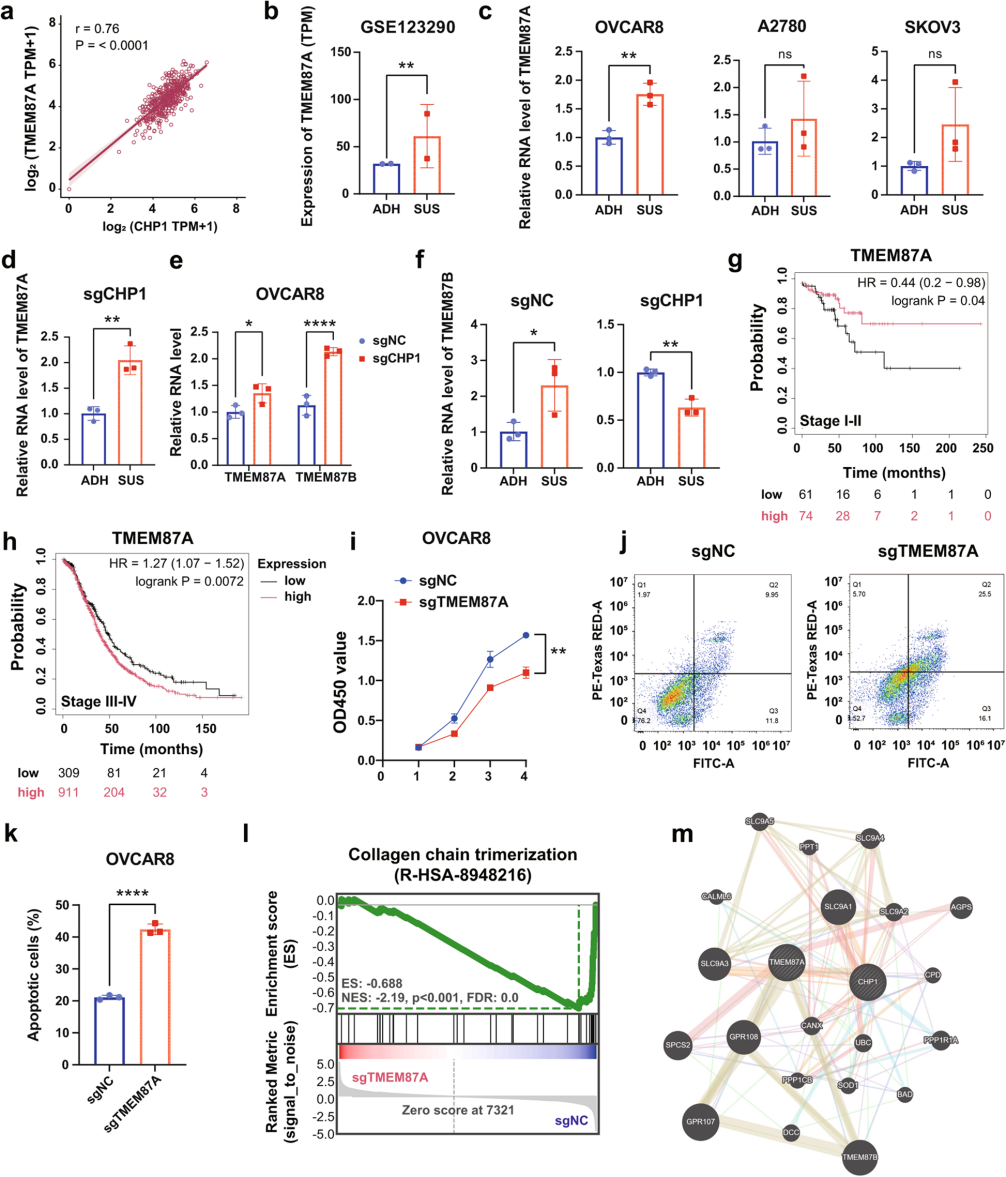
TMEM87A is a key interacting partner of CHP1 and shares its mechanosensitive phenotype. **a** Pearson’s correlation between *CHP1* and *TMEM87A* mRNA expression in ovarian cancer from GEPIA2 (data source: TCGA-OV). **b ***TMEM87A* expression (TPM) in adherent vs. suspended cells from GSE123290. **c** RT-qPCR analysis of *TMEM87A* mRNA expression in adherent vs. suspended OVCAR8, A2780, and SKOV3 cells (n = 3 independent experiments). **d** RT-qPCR analysis of *TMEM87A* mRNA expression in adherent vs. suspended OVCAR8-*sgCHP1* cells (n = 3 independent experiments). **e** RT-qPCR analysis of *TMEM87A* and *TMEM87B* mRNA expression in OVCAR8-*sgNC* and *sgCHP1* cells (n = 3 independent experiments). **f** RT-qPCR analysis of *TMEM87B* mRNA expression in adherent vs. suspended OVCAR8-*sgNC* and *sgCHP1* cells (n = 3 independent experiments). **g**–**h** Kaplan–Meier survival analysis of *TMEM87A* expression in stage I–II (**g**) and stage III–IV (**h**) ovarian cancer patients. **i** CCK-8 proliferation assay of OVCAR8-*sgNC* and *sgTMEM87A* cells (n = 3 independent experiments). **j** Representative flow cytometry plots (Annexin V/PI staining) of OVCAR8-*sgNC* and *sgTMEM87A* cells under suspension conditions. **k** Quantification of apoptotic cells (%) in OVCAR8-*sgNC* and *sgTMEM87A* cells under suspension conditions (n = 3 independent experiments). **l** GSEA plot showing suppression of collagen chain trimerization in *sgTMEM87A* vs. *sgNC* cells. **m** GeneMANIA network demonstrating the predicted interaction between TMEM87A and CHP1. * *P* < 0.05; ** *P* < 0.01; *** *P* < 0.001; **** *P* < 0.0001

CHP1 functions as a molecular chaperone, and its role may depend on the proteins it binds to. Bioinformatic analysis via GEPIA2 identified *TMEM87A* as the top co-expressed gene with *CHP1* in ovarian cancer (Pearson r = 0.7; Fig. 3a). *TMEM87A* is a recently characterized mechanosensitive ion channel that shares phenotypic similarities with *sgCHP1* cells in spheroid formation, cell–cell interaction and cell–matrix interaction [11, 12]. *TMEM87A* expression was elevated upon suspension in OVCAR8 cells and in the GSE123290 database (Fig. 3b-c), mirroring the expression pattern of *CHP1*. *TMEM87A* was also elevated in OVCAR8-*sgCHP1* cells in response to suspension (Fig. 3d).

As TMEM87B can function redundantly with TMEM87A, the expression of both paralogs was analyzed upon *CHP1* knockout and suspension. *CHP1* knockout increased the expression of both *TMEM87A* and *TMEM87B*; however, unlike *TMEM87A*, *TMEM87B* expression increased in *sgCHP1* cells under adherent conditions but decreased upon suspension (Fig. 3e–f), suggesting a possible compensatory mechanism underlying the elevated *TMEM87A* expression in *sgCHP1* cells. *TMEM87A* high-expression correlated with better prognosis in early-stage patients (stage I–II; n = 135, HR = 0.44, logrank P = 0.04) but poorer outcomes in late-stage patients (stage III–IV; n = 1,220, HR = 1.27, logrank P = 0.0072; Fig. 3g–h). Consistent with *CHP1*, *TMEM87A* knockout also significantly reduced OVCAR8 cell proliferation (Fig. 3i), increased apoptosis under suspension (Fig. 3j–k), and suppressed collagen chain trimerization by GSEA (Fig. 3l). GeneMANIA datasets suggested a close interaction between TMEM87A and CHP1 (Fig. 3m). CHP1 was reported to be localized in the plasma and Golgi apparatus, while TMEM87A was mainly observed at the Golgi membrane and cell surface. Our immunofluorescence results surprisingly suggested that the overexpression of CHP1 and TMEM87A built synapse-like structure and transferred TMEM87A-EGFP signals among cells (Fig. S1f–g). In the previous studies, CHP1 (annotated as CHP in the original dataset) was captured in the protein identified in WM266-4, where it was believed that the existence of the protein, regardless of its expression, might influence mechanical-sensing or the function of TMEM87A [11, 12] (Fig. S1h).

### The CHP1-TMEM87A complex regulates spheroid morphogenesis and GPC6-WNT5A-mediated metastatic signaling

**Fig. 4:**
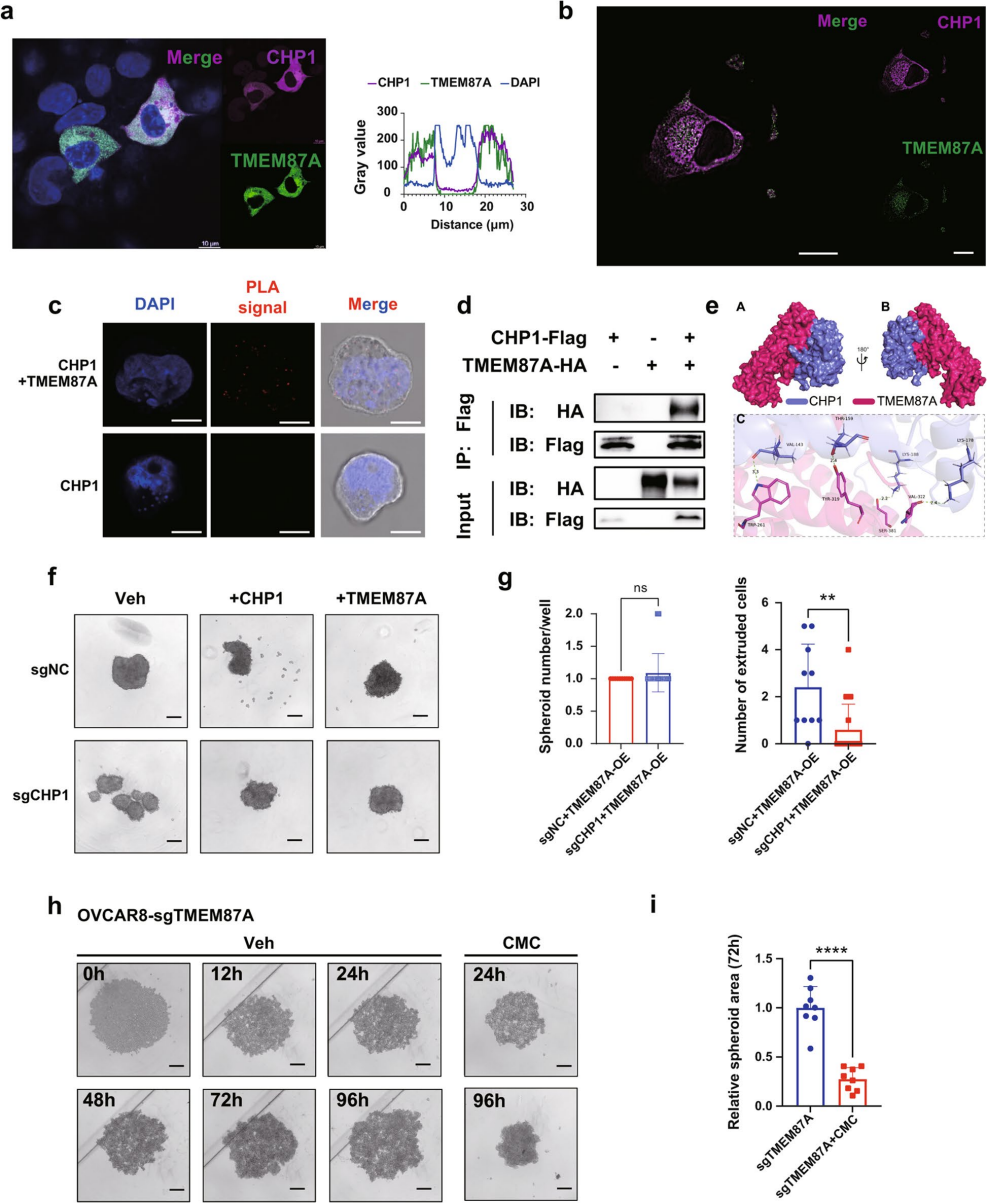
The CHP1-TMEM87A complex regulates spheroid morphogenesis. **a** Immunofluorescence (IF) staining of co-transfected CHP1-mCherry (purple) and TMEM87A-EGFP (green) in OVCAR8 cells, with line-scan co-localization analysis. Scale bar, 10 µm. **b** HIS-SIM super-resolution imaging of co-transfected CHP1-mCherry and TMEM87A-EGFP showing co-localization at synapse-like structures. Scale bar, 10 μm. **c** Proximity ligation assay (PLA) of CHP1-TMEM87A interaction in OVCAR8 cells. CHP1 alone serves as negative control. Scale bar, 5 μm. **d** Co-immunoprecipitation (co-IP) of exogenous CHP1-3 × Flag and TMEM87A-3 × HA in OVCAR8 cells. Single-tag transfections (CHP1-Flag alone or TMEM87A-HA alone) serve as negative controls (n = 3 independent experiments). **e** Protein–protein docking analysis of CHP1 (blue) and TMEM87A (pink), showing two orientations (A, B) and key interaction residues at the binding interface (C). **f** Representative images of spheroid formation in OVCAR8-sgNC and sgCHP1 cells with vehicle (Veh), *CHP1* overexpression (+ CHP1), or *TMEM87A* overexpression (+ TMEM87A). Scale bar, 50 μm. **g** Quantification of spheroid number per well (left) and number of extruded cells (right) in sgNC + *TMEM87A-OE* vs. *sgCHP1* + *TMEM87A-OE* rescue experiments (n = 12 wells per group). **h** Time-course images of OVCAR8-*sgTMEM87A* spheroid formation with vehicle (Veh) or CMC treatment at 0, 12, 24, 48, 72, and 96 h. Scale bar, 50 μm. **i** Quantification of relative spheroid area at 72 h for OVCAR8-*sgTMEM87A* with or without CMC treatment (n = 12 wells per group). Sanger sequencing and Western blot validation of *TMEM87A* knockout are shown in Fig. S1c. * P < 0.05; ** P < 0.01; *** P < 0.001; **** P < 0.0001

Co-transfection of CHP1-mCherry and TMEM87A-EGFP revealed partial co-localization, confirmed by HIS-SIM super-resolution imaging (Fig. 4a–b). Proximity ligation assay (PLA) with anti-CHP1 and anti-TMEM87A antibodies revealed specific punctate signals in the cytoplasm and plasma membrane, confirming proximity interaction between the two proteins (Fig. 4c). Exogenous Co-IP confirmed the interaction, and rigid-body docking (GRAMM) revealed a high-affinity complex (−541 kcal/mol) with critical hydrogen bonds at VAL_143_-TRP_261_, THR_159_-TYR_319_, LYS_188_-SER_381_, and LYS_178_-VAL_322_ (Fig. 4d–e). Overexpression of either CHP1 or TMEM87A rescued the altered spheroid formation caused by *CHP1* knockout, enabling formation of a single spheroid per well (Fig. 4f–g). *TMEM87A*-knockout OVCAR8 cells was generated with CRISPR/Cas9. Sanger sequencing confirmed successful ablation with a homozygous 14-bp frameshift deletion (non-triplet multiple), and the WB validated a complete depletion of TMEM87A (Fig. S1c). *TMEM87A*-knockout OVCAR8 cells exhibited a distinct phenotype of loose aggregates rather than compact spheroids, which was reconstituted by carboxymethyl cellulose (CMC) addition to increase medium viscosity (Fig. 4h–i).

**Fig. 5:**
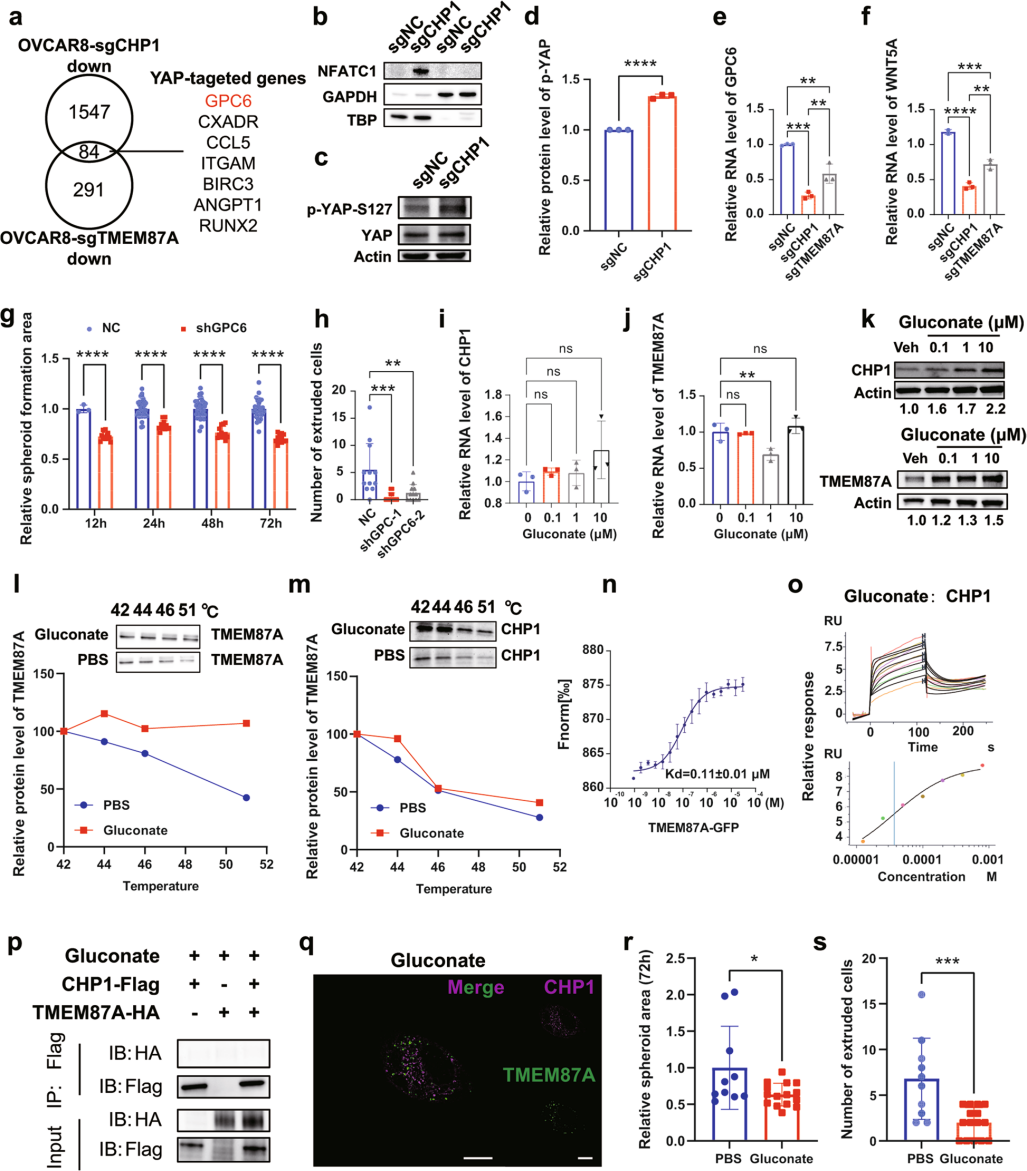
The CHP1-TMEM87A complex modulates GPC6-WNT5A to promote invasive migration and is disrupted by sodium gluconate. **a** Venn diagram demonstrating co-downregulated genes upon CHP1 and TMEM87A knockout, with enrichment for YAP-targeted genes. **b** Western blot of NFATC1 protein expression in OVCAR8-*sgNC* and *sgCHP1* cells. GAPDH serves as cytoplasmic loading control; TBP (TATA-binding protein) serves as nuclear loading control. **c**–**d** Western blot and quantification of p-YAP-Ser127 and total YAP in OVCAR8-*sgNC* and *sgCHP1* cells (n = 3 independent experiments). Actin serves as loading control. **e** RT-qPCR analysis of GPC6 mRNA expression in OVCAR8-*sgNC*, *sgCHP1*, and *sgTMEM87A* cells (n = 3 independent experiments). **f** RT-qPCR analysis of *WNT5A* mRNA expression in OVCAR8-*sgNC*, *sgCHP1*, and *sgTMEM87A* cells (n = 3 independent experiments). **g** Relative spheroid formation area of OVCAR8-NC and shGPC6 cells over time (12 h: n = 3; 24, 48, 72 h: NC n = 36, shGPC6 n = 12 wells per group). **h** Number of extruded cells in OVCAR8-NC and shGPC6 spheroids (n = 12 wells per group). **i** RT-qPCR analysis of *CHP1* mRNA expression upon sodium gluconate treatment (0, 0.1, 1, 10 µM) in OVCAR8 cells (n = 3 independent experiments). **j** RT-qPCR analysis of *TMEM87A* mRNA expression upon sodium gluconate treatment (0, 0.1, 1, 10 µM) in OVCAR8 cells (n = 3 independent experiments). **k** Western blot of CHP1 and TMEM87A protein expression upon sodium gluconate treatment at 0, 0.1, 1, and 10 µM for 48 h. Actin serves as loading control (n = 3 independent experiments). **l** Cellular thermal shift assay (CETSA) showing thermal stabilization of TMEM87A protein by sodium gluconate vs. PBS at 42, 44, 46, and 51 °C . **m** CETSA showing thermal stabilization of CHP1 protein by sodium gluconate vs. PBS at 42, 44, 46, and 51 °C. **n** Microscale thermophoresis (MST) binding curve of sodium gluconate with TMEM87A-EGFP (Kd = 0.11 ± 0.01 µM). **o** Surface plasmon resonance (SPR) sensorgrams showing binding of sodium gluconate to CHP1 protein. **p** Co-immunoprecipitation of CHP1-3 × Flag and TMEM87A-3 × HA in OVCAR8 cells treated with 1 µM sodium gluconate. Single-tag transfections serve as negative controls (n = 3 independent experiments). **q** HIS-SIM super-resolution imaging showing the co-localization of CHP1-mCherry and TMEM87A-EGFP upon sodium gluconate treatment. Scale bar, 10 μm. **r** Relative spheroid formation area at 72 h in OVCAR8 cells treated with PBS (n = 10) or sodium gluconate (n = 15). **s** Number of extruded cells in OVCAR8 spheroids treated with PBS (n = 10) or sodium gluconate (n = 15). * P < 0.05; ** P < 0.01; *** P < 0.001; **** P < 0.0001

Further analysis identified a set of co-downregulated genes upon *CHP1* and *TMEM87A* knockout, which was notably enriched for downstream target genes of the YAP signaling pathway (Fig. 5a). *CHP1* knockout led to increased NFATC1 protein expression and a relative increase in p-YAP-Ser127 (Fig. 5b-d), consistent with YAP inactivation. *GPC6* and *WNT5A* mRNA expression were significantly reduced upon knockout of either *CHP1* or *TMEM87A* (Fig. 5e–f). Functionally, *GPC6* knockdown markedly impaired spheroid formation and reduced cell extrusion suggesting that the CHP1-TMEM87A complex regulates Hedgehog and non-canonical WNT5A-mediated metastatic signaling (Fig. 5g-h; Fig. S1d-e).

To explore CHP1/TMEM87A-targeted anti-tumor strategies, sodium gluconate, a recently identified TMEM87A modulator (effective at 0.1 μM), has been evaluated for its therapeutic potential [11]. Sodium gluconate treatment did not significantly alter *CHP1* and *TMEM87A* mRNA expression at the tested concentrations (Fig. 5i–j), though a trend toward decreased expression was observed at higher doses. This suggests that the primary regulatory mechanism of sodium gluconate on the CHP1-TMEM87A complex is likely post-translational rather than transcriptional, consistent with its direct binding to TMEM87A protein (Fig. 5k). The upregulated level might be attributed to their binding to sodium gluconate, as confirmed by multiple techniques: cellular thermal shift assay (CETSA), microscale thermophoresis (MST; Kd = 0.11 ± 0.01 µM for TMEM87A), and surface plasmon resonance (SPR) showing binding of sodium gluconate to CHP1 (Fig. 5l–o). Importantly, 1 µM sodium gluconate disrupted the CHP1-TMEM87A protein interaction (Fig. 5p) and altered their co-localization, significantly reducing spheroid formation area and the number of extruded cells (Fig. 5p–s). This indicates that sodium gluconate likely suppresses the downstream oncogenic signaling by disrupting the CHP1-TMEM87A complex.

### CHP1/TMEM87A ablation suppresses tumor growth and low-dose sodium gluconate disrupts the complex for anti-tumor therapy

**Fig. 6:**
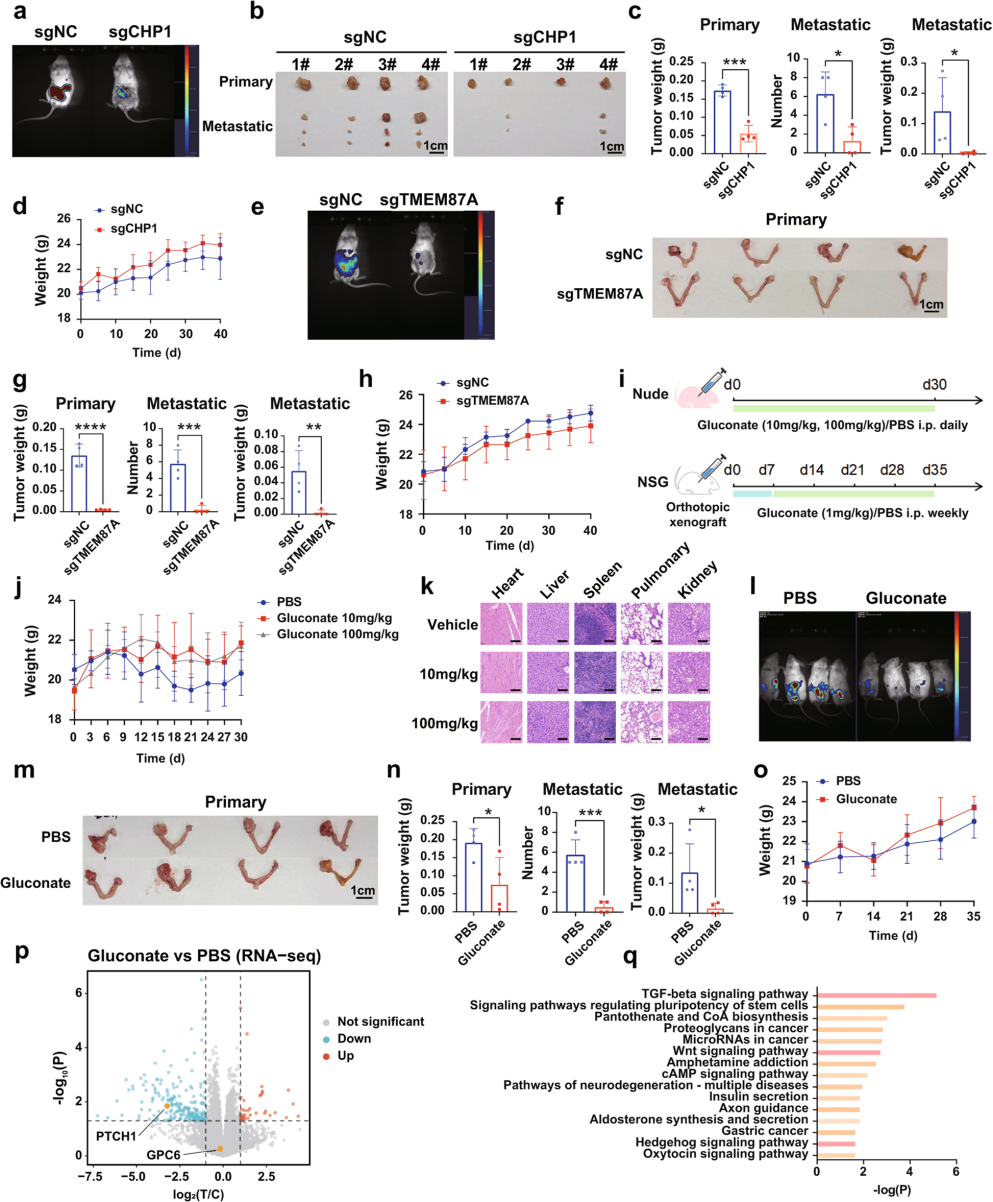
CHP1/TMEM87A ablation suppresses tumor growth and low-dose sodium gluconate disrupts the complex for anti-tumor therapy. **a** Representative in vivo bioluminescence images of orthotopic OVCAR8-*sgNC*-luc and *sgCHP1*-luc tumors in NSG mice. **b** Representative photographs of primary and metastatic tumors from *sgNC* and *sgCHP1* groups. Scale bar, 1 cm. **c** Quantification of primary tumor weight, metastatic tumor number, and metastatic tumor weight in *sgNC* vs. *sgCHP1* groups (n = 4 mice per group). **d** Mouse body weight changes throughout the treatment period in *sgNC* vs. *sgCHP1* groups (n = 4 mice per group). **e** Representative in vivo bioluminescence images of orthotopic OVCAR8-*sgNC*-luc and *sgTMEM87A*-luc tumors. **f** Representative photographs of primary tumors from *sgNC* and *sgTMEM87A* groups. Scale bar, 1 cm. **g** Quantification of primary tumor weight, metastatic tumor number, and metastatic tumor weight in *sgNC* vs. *sgTMEM87A* groups (n = 4 mice per group). **h** Mouse body weight changes throughout the treatment period in *sgNC* vs. *sgTMEM87A* groups (n = 4 mice per group). **i** Schematic diagram of the sodium gluconate administration regimen: toxicology study in nude mice (10 or 100 mg/kg, i.p. daily for 30 days) and therapeutic study in NSG mice (1 mg/kg, i.p. weekly). **j** Mouse body weight changes during the toxicology study (PBS, 10 and 100 mg/kg gluconate groups). **k** H&E staining of major organs (heart, liver, spleen, lung, and kidney) from PBS and sodium gluconate-treated mice. Scale bar, 200 μm. **l** Representative in vivo bioluminescence images of orthotopic OVCAR8-*sgNC*-luc tumors treated with PBS or sodium gluconate (1 mg/kg, weekly). **m** Representative photographs of primary tumors from PBS and gluconate groups. Scale bar, 1 cm. **n** Quantification of primary tumor weight, metastatic tumor number, and metastatic tumor weight in PBS vs. gluconate groups (n = 4 mice per group). **o** Mouse body weight changes throughout the therapeutic study in PBS vs. gluconate groups (n = 4 mice per group). **p** Volcano plot showing differentially expressed genes in gluconate-treated vs. PBS-treated OVCAR8 cells (RNA-seq; up: 34, down: 196). **q** KEGG pathway enrichment analysis of downregulated genes upon gluconate treatment. * *P* < 0.05; ** *P* < 0.01; *** *P* < 0.001; **** *P* < 0.0001

Orthotopic implantation of luciferase-tagged OVCAR8 cells into the ovarian fat pad of NSG mice revealed significantly reduced primary tumor growth and metastasis in *sgCHP1*-luc and *sgTMEM87A*-luc groups, while body weight measurements showed no significant differences among the groups (Fig. 6a–h). Sodium gluconate administration at up to 100 mg/kg i.p. daily for 30 days was well tolerated, with no visceral toxicity by histological examination and no significant body weight changes (Fig. 6i–k). Low-dose sodium gluconate (1 mg/kg, i.p. weekly) significantly suppressed tumor growth and metastasis in OVCAR8 orthotopic models (Fig. 6l–m). Mouse body weights were monitored throughout the treatment period and showed no significant differences between treatment groups (Fig. 6n-o). RNA-seq analysis of gluconate-treated OVCAR8 cells identified 34 upregulated and 196 downregulated genes; KEGG pathway enrichment revealed significant suppression of the Hedgehog signaling pathway (Fig. 6p-q). These results position low-dose gluconate as a promising therapeutic strategy for CHP1/TMEM87A-driven tumors.

## Discussion

Our mechanistic studies suggest that the CHP1-TMEM87A complex governs cohesion, spheroid formation, and mechanosensing in HGSC cells. Knockout of either component induced spheroid fragmentation and enhanced ECM adhesion, phenotypes associated with *GPC6* and *WNT5A* downregulation, and reduced tumor growth and metastasis in vivo. Notably, our in vivo data indicate that the CHP1-TMEM87A complex contributes to overall tumor progression, encompassing both primary tumor growth and metastatic dissemination, rather than being exclusively metastasis-specific. CHP1 is often regarded as a chaperone that supports the function of its interacting proteins. Meanwhile, TMEM87A is a lately identified mechanosensitive channel [12], and proteomic analysis has shown that low *TMEM87A* expression is associated with favorable prognosis in endometrioid ovarian carcinoma [30]. Most importantly, our results suggest that the function of TMEM87A is largely dependent on its interaction with CHP1, bringing new insight into the potential modulation between cellular pH homeostasis and mechanical stress, as hinted at by the only existing study on TMEM87A in cancer [12]. Although not directly confirmed in this study, we propose that CHP1 might be an important regulator linking cellular pH homeostasis and mechanical stress. Notably, our in vivo data indicate that the CHP1-TMEM87A complex contributes to overall tumor progression, encompassing both primary tumor growth and metastatic dissemination. While our in vitro data emphasize the complex’s role in anchorage-independent survival and spheroid integrity, processes most directly relevant to metastasis, the orthotopic model does not permit clean dissociation of proliferative and metastatic effects. We therefore explicitly acknowledge that CHP1 and TMEM87A may support tumor progression through multiple mechanisms beyond metastasis per se, and that the complex may represent a broader vulnerability in ovarian cancer biology.

Recently, Mitchell et al. [31] proposed that the extrusion of epithelial cells under crowded conditions is determined by epithelial tension or their bioenergetic fitness. They demonstrated that crowding activates the epithelial sodium channel (ENaC), leading to sodium influx and membrane depolarization. Cells with sufficient adenosine triphosphate (ATP) can repolarize via the Na^+^/K^+^ ATPase, whereas energetically compromised cells remain depolarized, triggering potassium efflux through voltage-gated K^+^ channels, water loss, and eventual extrusion. This mechanism offers a novel conceptual framework for understanding the function of the CHP1-TMEM87A complex. Our work identifies CHP1-TMEM87A as another potential mechanosensitive signaling hub. Given the well-established role of CHP1 as a critical cofactor for the NHE1, which collaborates with the Na^+^/K^+^ ATPase to maintain ionic and pH homeostasis, we speculate that the CHP1-TMEM87A complex may sense crowding stress in a manner analogous to ENaC, though direct experimental validation of this mechanism will be required in future studies. In energetically competent cells, downstream signaling may be effectively buffered, whereas under bioenergetic deficiency, sustained CHP1-TMEM87A activation may promote extrusion and cell death via pathways such as non-canonical WNT5A signaling.

This bioenergetics-based model of cell fate selection provides a plausible explanation for the stage-dependent prognostic significance of *CHP1/TMEM87A* in ovarian cancer. In early-stage tumors, high *CHP1/TMEM87A* expression may exert a tumor-suppressive role by eliminating fewer fit cells. In contrast, within the harsh tumor microenvironment of advanced disease, this pathway may be hijacked by cancer cells to facilitate detachment and dissemination, thereby promoting metastasis and correlating with poor outcomes.

The downstream signaling axis identified in our study, is supported by a growing body of independent evidence. YAP/TAZ, the transcriptional effectors of the Hippo pathway, are well-established sensors of mechanical cues and are pervasively activated across solid tumors to drive proliferation, EMT, and metastasis [32, 33]. In HGSC specifically, YAP activity has been linked to cellular plasticity and peritoneal dissemination through the ET-1/ZEB1/YAP axis [34], and mechanosensitive channels such as PIEZO1 have been shown to promote ovarian cancer metastasis via Hippo/YAP signaling [35], providing a precedent for mechanosensitive channel-YAP crosstalk of the type we propose for TMEM87A. Regarding *GPC6*, prior work in breast carcinoma demonstrated that NFAT transcriptionally induces *GPC6*, which in turn activates WNT5A signaling to drive invasive migration [36]. This NFAT-GPC6-WNT5A axis is particularly relevant to our findings given that NFATC1 protein expression was elevated upon *CHP1* knockout in our study, suggesting a potential compensatory or co-regulatory relationship between the CHP1-TMEM87A complex and NFAT-mediated transcription. At the level of WNT5A, multiple lines of evidence support its oncogenic role in ovarian cancer: WNT5A is enriched in malignant ascites and promotes tumor cell adhesion to peritoneal mesothelial cells and directional migration [37, 38], while WNT5A-ROR2 signaling has been shown to drive early mesothelial invasion [38]. Notably, WNT5A and TGFβ1 have been reported to converge through YAP1 activity to promote EMT and mesothelial activation in ovarian cancer cells [39], providing a mechanistic link between the YAP activity changes we observe and the downstream WNT5A-mediated metastatic phenotype. Finally, the spheroid integrity defects caused by CHP1-TMEM87A loss are consistent with the established role of multicellular spheroid cohesion in anoikis resistance and transcoelomic dissemination in HGSC [4]. Collectively, these published findings lend contextual support to the pathway we propose, though direct mechanistic connections between each node remain to be experimentally validated in future work.

Additionally, we propose a potential drug repurposing strategy using sodium gluconate to target the CHP1-TMEM87A complex. Sodium gluconate is a quite ancient drug believed to have little toxicity. Previous investigations into its anti-cancer potential were primarily confined to colon cancer, where dietary administration at an extremely high dose (50 g GNA/kg basal diet) was shown to reduce tumor incidence in rats. This effect, however, was largely attributed to an indirect mechanism involving increased butyrate production [40, 41]. In stark contrast to this high-dose approach, our study explores a low-dose regimen, guided by the specific molecular target TMEM87A. We show that low-dose sodium gluconate disrupts the CHP1-TMEM87A interaction, suggesting that its antitumor effect may arise from direct interference with this mechanosensitive complex rather than from indirect metabolic effects. The previously reported high-dose dietary effects should not be directly extrapolated to the mechanism proposed here, as such high doses may not only be ineffective but could potentially exert opposing effects by overwhelming the specific therapeutic blockade of the CHP1-TMEM87A function.

Although our study suggests that the CHP1-TMEM87A complex may serve as a key regulator of HGSC metastasis, several limitations should be acknowledged. First, we used human-derived cell lines in an immunosuppressive model, so we could not investigate the function of CHP1-TMEM87A and the anti-tumor effect of gluconate in the presence of immune cells in this study. Additionally, the majority of our in vitro experiments were performed in OVCAR8 cells, a well-established HGSC model. While we also observed CHP1 expression changes in A2780 (endometrioid subtype) and SKOV3 (adenocarcinoma) cells, the differential responses across cell lines highlight the heterogeneity of ovarian cancer subtypes, and the generalisability of our findings to other HGSC models warrants further investigation. Furthermore, no direct mechanical stimulation experiments (e.g., fluid shear stress, substrate stiffness) were performed in this study; such experiments will be required to formally establish the mechanosensing function of the CHP1-TMEM87A complex. Finally, multivariate Cox regression analysis adjusting for clinical covariates (stage, grade, BRCA status) was not performed, which limits the strength of the prognostic conclusions. Future studies should directly investigate the interplay between cellular ATP levels, membrane potential, and activation of this complex, which will be essential for elucidating the mechanisms governing epithelial cell fate and developing novel anti-metastatic strategies.

## Conclusion

**Fig. 7:**
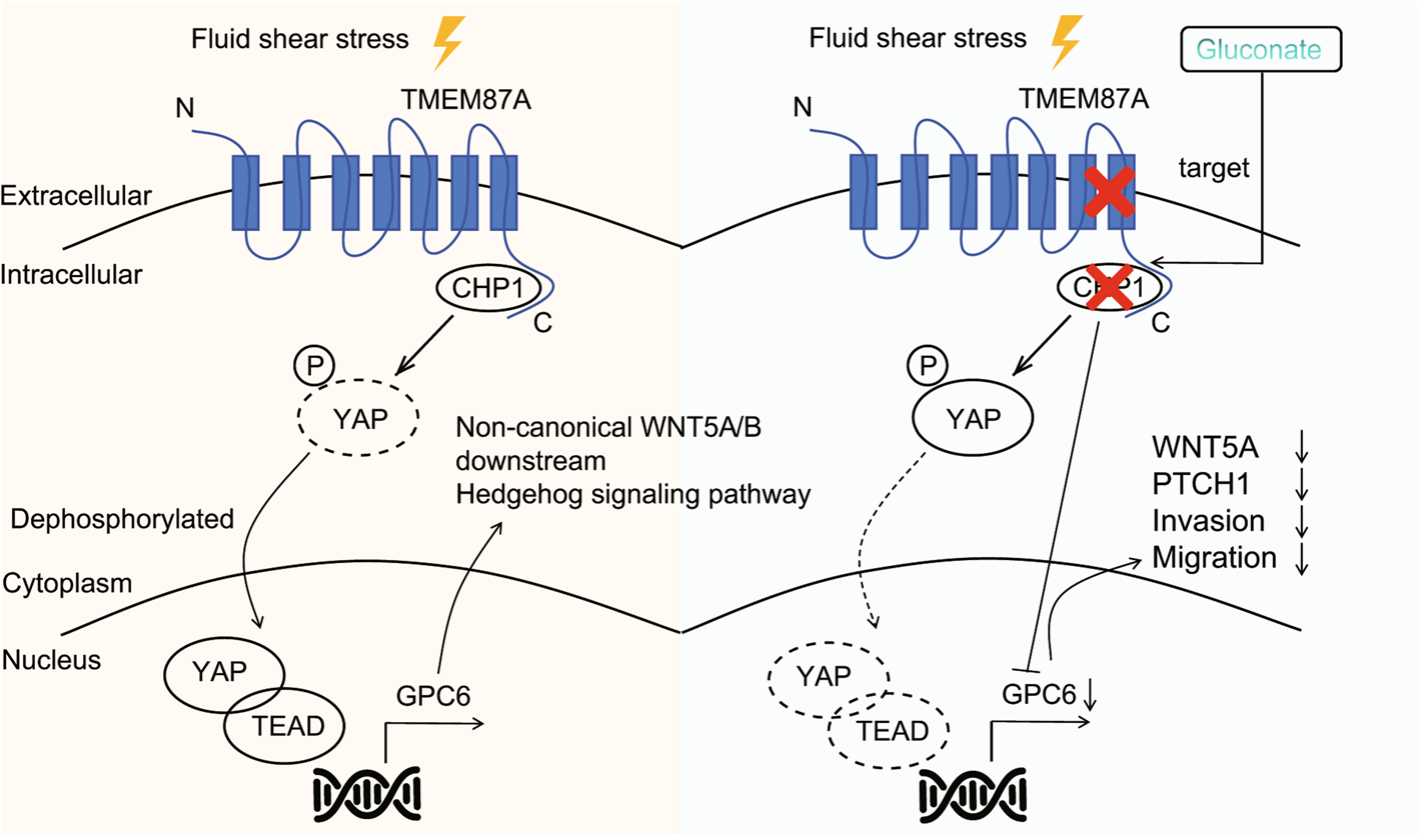
Working model of the CHP1-TMEM87A complex in modulating invasion and migration via GPC6-WNT5A signaling. The CHP1-TMEM87A putative mechanosensing hub promotes HGSC spheroid integrity and metastasis by sustaining YAP activity and downstream GPC6-WNT5A/Hedgehog signaling. Knockout of CHP1 or TMEM87A disrupts complex formation, leading to YAP inactivation (increased p-YAP-Ser127), downregulation of GPC6 and WNT5A, impaired spheroid cohesion, and reduced metastatic capacity. Sodium gluconate binds directly to TMEM87A and CHP1, disrupts the complex, and phenocopies genetic ablation, suppressing tumor growth and metastasis in vivo

In summary, this study demonstrates CHP1’s role in spheroid formation and ovarian cancer metastasis, and identifies the CHP1-TMEM87A complex as a therapeutically targetable mechanotransduction hub driving HGSC progression. Low-dose sodium gluconate significantly suppresses in vivo tumor growth and extends survival without causing visceral toxicity, while genetic disruption of CHP1-TMEM87A impairs metastasis via GPC6-WNT5A pathway inhibition. These findings offer two actionable strategies, direct targeting of the CHP1-TMEM87A interface, and clinical translation of low-dose gluconate to overcome cancer metastasis (Fig. 7).

## Materials and methods

### Cell culture and transfection

Ovarian cancer cell (A2780, OVCAR8, SKOV3) and HEK293FT were brought from Wuhan Pricella Biotechnology Co., Ltd or Cell Bank, Chinese Academy of Sciences. OVCAR8 was cultured in Roswell Park Memorial Institute 1640 (RPMI-1640) medium (Gibco, 11,875,119), while other cells were cultured in Dulbecco’s modified eagle’s medium (DMEM) (Corning, 10–013-CV), supplemented with 100 μg/mL streptomycin, 100 IU/mL penicillin (Macklin, Q6532) and 10% fetal bovine serum (FBS) (XY CELL, Cat# SR01C-500), at 37°C in a humidified atmosphere containing 5% CO_2_. Cell lines were authenticated using short tandem repeat (STR) profiling and confirmed to be free of mycoplasma contamination. For generation of cell lines derived organoids, cells were mixed with Matrigel (Corning, 356231), plated into 96-well plates and cultured in OC organoid medium (bioGenous, K2168).

*CHP1* and *TMEM87A* knockout in OVCAR8 cells was achieved using CRISPR/Cas9. sgRNA (5'−3' AAATCACTCGCCTCTACAGCCGG) for CHP1 and sgRNA for TMEM87A (5'−3' TGAATACTCACCGACGGTATCGG) was cloned into Lenti-cas9, validated by sequencing, and packaged into lentivirus (Gag-Pol/pCMV-VSVG co-transfection in HEK293FT cells), using Lipo8000 transfection reagent (Beyotime, C0533). OVCAR8 cells were infected [(MOI = 5; 6 µg/mL polybrene (Yeasen, 40804ES76)] and selected with puromycin (Amresco, J593). Monoclonal lines were subsequently isolated and expanded.

Knockdown of *CHP1*, and overexpression of *CHP1* and *TMEM87A* was achieved with lentiviral shRNAs targeting *CHP1*, *TMEM87A*, and pLV3-CMV-CHP1-3xFlag-copGFP, pCMV-CHP1-mcherry, pLV3-CMV-TMEM87A-3xHA-mcherry, pCMV-TMEM87A-EGFP overexpression plasmid, along with control EGFP or mcherry plasmids. The construction of the plasmid was performed by Miaolingbio. Positive cells were selected using puromycin. OVCAR8-*sgNC*, OVCAR8-*sgCHP1*, OVCAR8-*sgTMEM87A*-luc cells were generated by transfecting OVCAR8 and its derivatives with a luciferase-mcherry reporter plasmid.

### Suspension culture and generation of spheroid

Cells were cultured in suspension using ultra-low attachment (ULA) 96-well, 24-well, 6-well plates (Corning, 7007/3473/3471) and cell spheroid honeycomb culture chip (Vivoid, AUT042801) for spheroid formation assays. For generation of mosaic spheroid, the transfected cells were first selected by flow cytometry sorting (FACS) to achieve purity and cultured for 1–2 passages to maintain a good condition. According to the cell counts, cells were mixed and plated into ULA 96-well plates and centrifuged. The growth of spheroid was monitored and recorded.

### Cell viability assay

Cell viability was assessed using the Cell Counting Kit-8 (CCK-8; targetmol, C0005ED) assay. Cells were seeded at a density of 3,000 cells per well in 96-well plates and cultured under standard conditions. At 24, 48, 72, 96, and 108 h after seeding, CCK-8 reagent (10 μL per well) was added and cells were incubated at 37 °C for 2 h. Absorbance was measured at 450 nm using a microplate reader. Each condition was performed in triplicate and experiments were repeated at least three times independently.

### Apoptosis assay

Apoptosis was assessed by Annexin V-FITC/propidium iodide (PI) staining using the Annexin V-FITC Apoptosis Detection Kit (Beyotime, C1062) according to the manufacturer’s instructions. Briefly, cells were cultured under suspension conditions for 48 h, then collected by centrifugation, washed twice with cold PBS, and resuspended in binding buffer. Cells were incubated with Annexin V-FITC and PI for 15 min at room temperature in the dark. Apoptotic cells were quantified by flow cytometry (LSRFortessa X-20, and data were analyzed using FlowJo software (v10).

### Migration and invasion assay

Cell migration and invasion were assessed using Transwell chambers (8-μm pore size; Corning). For invasion assays, the upper surface of the membrane was pre-coated with Matrigel (corning, 356,231) diluted in serum-free medium. Cells (5 × 10^4^ per well) were seeded in the upper chamber in serum-free medium, and complete medium containing 10% FBS was added to the lower chamber as a chemoattractant. After incubation for 30 h (migration) or 48 h (invasion) at 37 °C, non-migrated cells on the upper surface were removed with a cotton swab. Cells that had migrated to the lower surface were fixed with 4% paraformaldehyde, stained with 0.1% crystal violet, and counted in five random fields per well under a light microscope.

### Quantitative reverse transcription polymerase chain reaction (RT-qPCR)

RT-qPCR was performed using super fastpure cell RNA isolation kit (Vazyme, RC102-01) and (LABLEAD, R0202) according to the manufacturer’s protocol. Primer sequences were list in Table S2.

### Western blotting (WB)

Cells were lysed in High KCl buffer supplemented with protease/phosphatase inhibitors (Bimake, B14001/B15001) and sonicated as described before [42]. Protein concentrations were determined by BCA assay (Beyotime, P0012). Lysates were resolved by SDS-PAGE and transferred to PVDF membranes. Membranes were blocked with 5% skim milk or blocking buffer (Beyotime, P0252), followed by incubation with primary antibodies overnight at 4 °C or 1 h at RT. After six 5-min TBST washes, membranes were incubated with HRP-conjugated secondary antibodies. Signals were developed using ECL substrate (biosharp, BL523B) and imaged with a Bio-Rad ChemiDoc system. Band density was quantified using ImageJ2. Primary antibodies used for WB included anti-TMEM87A (Novus Biologicals, MAB7966, 1:2500), anti-CHP1 (GeneTex, GTX113936, 1:1000), anti-Actin (Epizyme, LF201S, 1:5000), anti-YAP (ABclonal, A19134, 1:20,000), anti-p-YAP-S127 (ABclonal, AP1436, 1:5000), anti-NFATC1 (ABclonal, A19597, 1:1000), anti-GPC6 (ABclonal, A2741, 1:1000), anti-GAPDH (Epizyme, LF205, 1:5000), anti-TBP (Proteintech, 22,006–1-AP, 1:1000), and anti-Flag tag (Abmart, R20008; 1:2000), anti-HA tag (Abmart, M20003, 1:5000). HRP-conjugated secondary antibodies (Abmart, M21001S; Abmart, M21002S) were used at 1:5000.

### Cellular thermal shift assay (CETSA)

CETSA was performed to assess the thermal stabilization of CHP1 and TMEM87A upon sodium gluconate treatment. Briefly, OVCAR8 cells from four 60-mm dishes were harvested, washed with PBS, and lysed in Co-IP lysis buffer (Proteintech, PR20037) supplemented with protease and phosphatase inhibitors on ice for 15 min. Clarified lysates (10,000 × g, 5 min, 4°C) were divided into two 700-μL aliquots and treated with either sodium gluconate (10 μM final) or PBS vehicle at 37 °C for 1.5 h. Each aliquot was then split into fractions and heated at 42, 44, 46, or 51 °C for 3 min, immediately chilled on ice, and re-equilibrated at room temperature for 3 min. After centrifugation (15,000 × g, 40 min, 4 °C), supernatants were collected. For TMEM87A detection, samples were denatured in 5 × loading buffer at room temperature for 30 min; for CHP1 detection, denaturation was performed at 95 °C for 5 min. Subsequently, samples were resolved by SDS-PAGE followed by membrane transfer.

### Co-immunoprecipitation (co-IP)

Magnetic Protein A + G beads (Beyotime, P2108M) were blocked with 1% BSA-PBS for 2 h at 4 °C to reduce non-specific binding. Cells were lysed in specialized Co-IP buffer (Proteintech, PR20037) supplemented with protease/phosphatase inhibitors. Lysates were centrifuged (10,000 g, 5 min, 4 °C), and supernatants were quantified by BCA assay. For pre-clearing, lysates (1 mg protein) were incubated with 20 μL beads for 20 min at 4 °C. Meanwhile, 500 μL TBST contained 2.5μL calcium binding protein p22 antibody (GeneTex, GTX113936) and Rabbit IgG (Beyotime, A7016) for endogenous co-IP, or 2.5μL anti-DYKDDDDK antibody (Abmart, R20008) for exogenous co-IP was conjugated to BSA-blocked beads (20 μL) by rotation (1 h, RT). Pre-cleared lysates were incubated with antibody-bound beads overnight at 4 °C with rotation. Beads were washed PBST, and bound proteins were eluted in 1 × loading buffer (95 °C, 5 min). Eluates were analyzed by WB.

### Proximity ligation assay (PLA)

PLA was performed using the NaveniFlex Cell Red Kit (Navinci) following the manufacturer’s instructions with minor modifications [43, 44]. In brief, cells were fixed in 4% paraformaldehyde, subjected to heat-mediated antigen retrieval, and permeabilized with 0.1% Triton X-100. After blocking, cells were incubated with mouse anti-CHP1 (OriGene, TA810226; 1:400) and rabbit anti-TMEM87A (Novus, NBP1-90,532; 1:50), or with anti-CHP1 alone for negative controls. Navenibody probes were then applied, followed by ligation and rolling circle amplification. Nuclei were counterstained with DAPI, and slides were mounted in antifade medium. Images were acquired on a Leica TCS SP8 confocal microscope using appropriate excitation/emission settings for DAPI and Texas Red.

### Immunofluorescence (IF) and immunohistochemistry (IHC) staining

For cell staining, cells were plated onto glass slides placed in 24-well plates, wrapped with Poly-D-lysine (Beyotime, ST508). Cells were fixed by 4% paraformaldehyde (PFA) for 10 min and then iced methanol for 10 min, blocked with 1% BSA (30 min, RT) and stained with mouse anti-CHP1 (OriGene, TA810226; 1:400) or rabbit anti-TMEM87A (Novus, NBP1-90,532; 1:200) according to the experiment requirements, and DAPI. For spheroid staining, spheroids around 150 μm were harvested and embedded in 3% low-melting-point agarose, fixed overnight in 4% PFA at 4 °C, paraffin-embedded, and sectioned (4 μm) for staining. Imaging was acquired using the 63 ×/1.5 NA oil immersion objective with Olympus SpinSR10 spinning disk confocal super resolution microscope.

### High intelligent and sensitive SIM (HIS-SIM) imaging

Super-resolution imaging of co-localization of CHP1 and TMEM87A was performed using HIS-SIM provided by CSR Biotech (Guangzhou) Co., Ltd. Images were acquired using a 100 ×/1.5 NA oil immersion objective (Olympus). SIM images were collected and analyzed as described previously [45]. Sparse deconvolution was carried out to further improve the image quality [46].

### Microscale thermophoresis (MST) binding assay

Cells were transfected with the TMEM87A-EGFP plasmid for 48 h, lysed with Co-IP buffer (Proteintech, PR20037) on ice for 15 min and then centrifuged at 10,000 g for 5 min at 4°C. The clarified supernatant was collected and used directly for the MST assay [47]. The lysate containing the EGFP-fusion protein was mixed with a 16-point, 1:1 serial dilution of gluconate sodium in assay buffer (50 mM HEPES, pH 7.4, 0.05% Tween-20). The mixtures were loaded into standard capillaries after a 20-min incubation at room temperature. Thermophoresis was measured using a NanoTemper Monolith X, and the dissociation constant (Kd) was determined using the integrated analysis software.

### Surface plasmon resonance (SPR) assay

Protein of CHP1 (expressed in mammalian cell) was brought from Cusabio (CSB-MP860773HU). For the interaction with protein and sodium gluconate, the ligand proteins CHP1 were diluted to a concentration of 15 μg/mL in a sodium acetate buffer (pH 4.0) for the protein immobilization procedure. The ligand immobilization method was designed with the following parameters: the sensor chip was activated for 7–10 min, followed by ligand coupling for 1200 s, and then the chip was blocked for 7 min to complete the immobilization process. The analyte, sodium gluconate, was prepared at a top concentration of 1.6 mM and subjected to 1:1 serial dilution, generating a total of eight concentration points. A multi-cycle kinetics run was performed using PBS-Tween-20 (0.05%) as the running buffer, with the parameters set to an association phase of 120 s and a dissociation phase of 120 s for each cycle. The raw data were imported into the Biacore™ Insight Evaluation Software (version 5.0.18.22102). The multi-cycle kinetics analysis method (Evaluation of multi-cycle kinetics for LMW samples, to calculate kinetic rate constants) was selected. A minimum of five consecutive analyte concentration points were chosen for global fitting using a 1:1 binding model. This fitting procedure yielded the association rate constant (ka), kd, and the equilibrium dissociation constant (KD).

### Protein docking

Protein crystal structures of CHP1 (PDB ID: 2E30) and TMEM87A (PDB ID: 8CTJ) were retrieved from the Protein Data Bank (PDB). Since no structure for TMEM87B was available in PDB, its predicted structure (Uniprot ID: Q96K49) was obtained from the AlphaFold Database. Rigid-body protein docking was subsequently performed using GRAMM-X (http://gramm.compbio.ku.edu/) to investigate the interaction between CHP1 and TMEM87A/TMEM87B. Final 3D structural representations were generated using PyMOL v2.6 [48, 49].

### Survival analysis

Kaplan–Meier survival analysis was performed using the KM plot online tool (https://kmplot.com/analysis/). Overall survival (OS) and progression-free survival (PFS) data for ovarian cancer patients were stratified by CHP1 and TMEM87A expression using the auto-select best cutoff option. Log-rank p-values and hazard ratios (HR) with 95% confidence intervals were reported. Only datasets with available follow-up data were included.

### Correlation analysis

Pearson’ s correlation analysis between gene expression levels was performed using the Gene Expression Profiling Interactive Analysis 2 (GEPIA2) online tool (http://gepia2.cancer-pku.cn/). The analysis utilized pre-processed RNA-seq expression data from The Cancer Genome Atlas (TCGA) Ovarian Cancer (OV) datasets, which are integrated within the GEPIA2 platform. TCGA data were accessed and analyzed through GEPIA2 in February 2025, in accordance with TCGA data access policies and GEPIA2’s terms of use. Correlation coefficients (R) and p-values were calculated and visualized as scatter plots.

### Statistical analysis

All statistical analyses were performed using GraphPad Prism 9.0. Data are presented as mean ± standard deviation (SD) from at least three independent experiments. Comparisons between two groups were performed using unpaired two-tailed Student’s t-test. Multiple group comparisons were performed using one-way analysis of variance (ANOVA) followed by Tukey’s post hoc test. A p-value of less than 0.05 was considered statistically significant.

### Animal models

OVCAR8-*sgNC*-luc, OVCAR8-*sgCHP1*-luc and OVCAR8-*sgTMEM87A*-luc cells (1 × 10^6^ cells) were injected orthotopically into the ovarian fat pad of 6-week-old NSG mice. For toxicology study, mice received intraperitoneal (i.p.) injections of either PBS or sodium gluconate (MCE, Cat# HY-B1092A) at doses of 1, 10, 20, 50, or 100 mg/kg every day for 30 days. For efficacy study, mice received i.p. injections of either PBS or sodium gluconate (1 mg/kg) once a week for 35 days.

## Supplementary Information

Supplementary Material 1: Tables S1 and S2. Fig. S1 Supplementary data for CHP1 and TMEM87A characterization. (a) Pearson’s correlation between *CHP1* expression and HA metabolism-associated genes (CD44, HAS2, HEXA, HMMR) in ovarian cancer from GEPIA2 (data source: TCGA-OV). (b) Sanger sequencing confirming homozygous 17-bp frameshift deletion in OVCAR8-*sgCHP1* cells, and Western blot validating complete depletion of CHP1 protein; Actin serves as loading control. (c) Sanger sequencing confirming homozygous 14-bp frameshift deletion in OVCAR8-*sgTMEM87A* cells, and Western blot validating complete depletion of TMEM87A protein; Actin serves as loading control. (d) Schematic diagram illustrating the CHP1-Calcineurin-NFAT signaling axis. (e) Schematic diagram of the proposed CHP1-TMEM87A-YAP-GPC6-WNT5A signaling pathway. (f) Live-cell intracellular imaging showing co-localization of CHP1 (red) and TMEM87A (green) at different time points (T3, T4, T5) during spheroid formation. Scale bar, 20 μm. (g) HIS-SIM super-resolution imaging showing the co-localization of CHP1 and TMEM87A-EGFP in synapse-like structures. Scale bar, 10 μm. (h) Venn diagram showing proteins identified in WM266-4 cells, with CHP1, GPC6, and YAP1 highlighted as co-identified proteins.

## Data Availability

The data supporting the findings of this study are available from the corresponding author upon reasonable request. RNA-seq data have been deposited in the Gene Expression Omnibus (GEO) under accession number GSE330905 (https://www.ncbi.nlm.nih.gov/geo/query/acc.cgi?acc=GSE330905). Sanger sequencing data generated in this study have been deposited in the NCBI Sequence Read Archive (SRA) under BioProject accession number PRJNA1465061 (SRA accession: SRP699536).
