# Supplementary material for: Targeting the Calcineurin Homologous Protein 1 (CHP1)-Transmembrane Protein 87A (TMEM87A) mechanosensing complex: a druggable vulnerability in metastatic ovarian cancer
Source: Mol Biomed. 2026 Jun 8;7:86. doi: 10.1186/s43556-026-00487-4 (PMC13247001; doi:10.1186/s43556-026-00487-4)
Supplement: Supplementary file 1 — Supplementary Material 1: Tables S1 and S2. Fig. S1 Supplementary data for CHP1 and TMEM87A characterization. (a) Pearson’s correlation between CHP1 expression and HA metabolism-associated genes (CD44, HAS2, HEXA, HMMR) in ovarian cancer from GEPIA2 (data source: TCGA-OV). (b) Sanger sequencing confirming homozygous 17-bp frameshift deletion in OVCAR8-sgCHP1 cells, and Western blot validating complete depletion of CHP1 protein; Actin serves as loading control. (c) Sanger sequencing confirming homozygous 14-bp frameshift deletion in OVCAR8-sgTMEM87A cells, and Western blot validating complete depletion of TMEM87A protein; Actin serves as loading control. (d) Schematic diagram illustrating the CHP1-Calcineurin-NFAT signaling axis. (e) Schematic diagram of the proposed CHP1-TMEM87A-YAP-GPC6-WNT5A signaling pathway. (f) Live-cell intracellular imaging showing co-localization of CHP1 (red) and TMEM87A (green) at different time points (T3, T4, T5) during spheroid formation. Scale bar, 20 μm. (g) HIS-SIM super-resolution imaging showing the co-localization of CHP1 and TMEM87A-EGFP in synapse-like structures. Scale bar, 10 μm. (h) Venn diagram showing proteins identified in WM266-4 cells, with CHP1, GPC6, and YAP1 highlighted as co-identified proteins. [file 43556_2026_487_MOESM1_ESM.docx]

**Supplementary Material**

**Title:** Targeting the Calcineurin Homologous Protein 1 (CHP1)–Transmembrane Protein 87A (TMEM87A) Mechanosensing Complex: A Druggable Vulnerability in Metastatic Ovarian Cancer

##### **Authors:** Ming-Zhu Jin^1, 2^, Heng-An Liu ^3^, Wen Di^1, 2, *^

##### **Affiliations:** ^1^ Department of Obstetrics and Gynecology, Renji Hospital, Shanghai Jiao Tong University School of Medicine, Shanghai, People's Republic of China

##### ^2^ Shanghai Key Laboratory of Gynecologic Oncology, Renji Hospital, Shanghai Jiao Tong University School of Medicine, Shanghai, People's Republic of China

##### ^3^ Mater Research Institute, The University of Queensland, Brisbane, QLD, Australia

**Corresponding author:**

Wen Di

Email: diwen163@163.com

**Tables**

## Supplemental Table S1. Hyaluronic acid (HA) metabolism-associated gene list and expression in ovarian cancer datasets.

| **Gene Symbol** | **UniProt ID** | **GSE123290 Log2FC** | **GSE123290 P-Value** | **GSE10971 logFC** | **GSE10971 Adj.P-Value** |
| --- | --- | --- | --- | --- | --- |
| *HAS2* | Q92819 | -6.7004 | 1.01e-04 | 9.3355 | 0.0436 |
| *CHP1* | Q99653 | 1.1641 | 0.0014 | 402.4858 | 2.66e-04 |
| *HMMR* | O75330 | -0.9739 | 0.0043 | 271.4126 | 1.12e-05 |
| *CD44* | P16070 | -1.7194 | 2.59e-04 | -39.4637 | 5.72e-04 |
| *HEXA* | P06865 | 0.7170 | 0.0337 | 25.1332 | 0.0272 |
| *HYAL3* | O43820 | 0.2349 | 0.6659 | 3.7458 | 0.0121 |
| *HYAL2* | Q12891 | 0.1351 | 0.7079 | 14.7622 | 0.0362 |
| *HEXB* | P07686 | 0.3303 | 0.3195 | 188.8319 | 0.0154 |
| *ABCC5* | O15440 | -0.1167 | 0.7264 | 927.8393 | 9.77e-06 |
| *STAB2* | Q8WWQ8 | -0.3755 | 0.7395 | ND | ND |
| *HYAL1* | Q12794 | 1.0902 | 0.3004 | ND | ND |
| *SLC9A1* | P19634 | 0.5383 | 0.1699 | ND | ND |
| *HAS3* | O00219 | -1.3651 | 0.0016 | ND | ND |
| *HAS1* | Q92839 | 2.0000 | 0.3712 | ND | ND |
| *GUSB* | P08236 | 0.4733 | 0.1339 | ND | ND |
| *CEMIP* | Q8WUJ3 | ND | ND | 25.6813 | 0.0263 |

## Supplemental Table S2. RT-qPCR primer sequences.

| **Gene name** | **Forward primer sequence** | **Reverse primer sequence** |
| --- | --- | --- |
| *CHP1* | CCAGAGGATTCCAGAACTTGCC | GGCGGAAATGAGCCAAAGTTCG |
| *TMEM87A* | GATTGGTGCTGTCATCTTCCTGG | TTCGAGCCAGTGAGCGTTTCAC |
| *TMEM87B* | GGGTACATCTCTGCATCAGACTG | CAGTAACAGGCAGACCACAGCA |
| *GPC6* | TATGGCTCTCCGTGTGATGACC | ACACGTCATCCATGCACCCACT |
| *WNT5A* | TACGAGAGTGCTCGCATCCTCA | TGTCTTCAGGCTACATGAGCCG |
| *PTCH1* | GCTGCACTACTTCAGAGACTGG | CACCAGGAGTTTGTAGGCAAGG |
| *IFNB1* | CTTGGATTCCTACAAAGAAGCAGC | TCCTCCTTCTGGAACTGCTGCA |
| *MID1* | TGAGTTCAGCGTGGTCTCCTAC | CACCGTGTAGTGGTTCTGCTTG |
| *EXPH5* | TGTCCCAAAGCCAGCAGTCATG | CTGAGCTTGCAGACTGTTCCTG |
| *ACTB* | CACCATTGGCAATGAGCGGTTC | AGGTCTTTGCGGATGTCCACGT |

# **Figures**

##
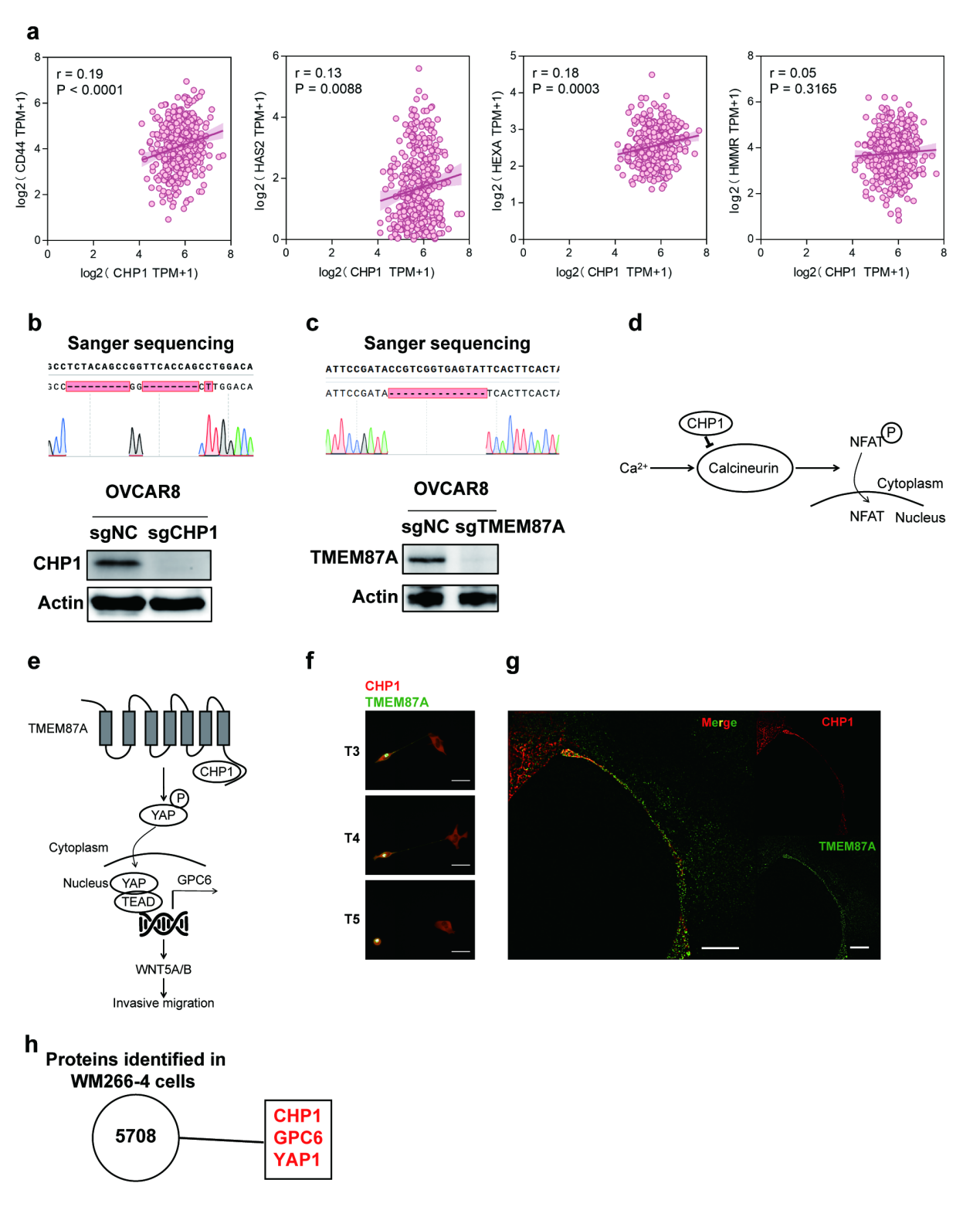
Supplementary Figure S1. VSupplementary data for CHP1 and TMEM87A characterization.

## (a) Pearson’s correlation between CHP1 expression and HA metabolism-associated genes (CD44, HAS2, HEXA, HMMR) in ovarian cancer from GEPIA2 (data source: TCGA-OV). (b) Sanger sequencing confirming homozygous 17-bp frameshift deletion in OVCAR8-sgCHP1 cells, and Western blot validating complete depletion of CHP1 protein; Actin serves as loading control. (c) Sanger sequencing confirming homozygous 14-bp frameshift deletion in OVCAR8-sgTMEM87A cells, and Western blot validating complete depletion of TMEM87A protein; Actin serves as loading control. (d) Schematic diagram illustrating the CHP1–Calcineurin–NFAT signaling axis. (e) Schematic diagram of the proposed CHP1–TMEM87A–YAP–GPC6–WNT5A signaling pathway. (f) Live-cell intracellular imaging showing co-localization of CHP1 (red) and TMEM87A (green) at different time points (T3, T4, T5) during spheroid formation. Scale bar, 20 μm. (g) HIS-SIM super-resolution imaging showing the co-localization of CHP1 and TMEM87A-EGFP in synapse-like structures. Scale bar, 10 μm. (h) Venn diagram showing proteins identified in WM266-4 cells, with CHP1, GPC6, and YAP1 highlighted as co-identified proteins.
